# People with dementia and family carers are welcoming of a model of dementia palliative care, but sceptical of its implementation

**DOI:** 10.1177/14713012241270777

**Published:** 2024-08-09

**Authors:** Siobhan Fox, Mary Faherty, Jonathan Drennan, Suzanne Guerin, W George Kernohan, Aileen Murphy, Suzanne Timmons

**Affiliations:** Centre for Gerontology and Rehabilitation, 8795 University College Cork, Ireland; Centre for Gerontology and Rehabilitation, School of Medicine, 8795 University College Cork, Ireland; School of Nursing and Midwifery, 8795 University College Cork, University College Dublin, Ireland; School of Psychology, 8797 University College Dublin, Ireland; Institute of Nursing and Health Research, 2596 Ulster University, Ireland; Department of Economics, 8795 University College Cork, Ireland; Centre for Gerontology and Rehabilitation, School of Medicine, 8795 University College Cork, Ireland

**Keywords:** dementia, palliative care, advance care planning, end-of-life, home care, community care, model

## Abstract

**Introduction:**

A palliative care approach can improve quality-of-life for people with dementia. It is the preference of many people with dementia to remain living at home until death, with the appropriate care. To develop a successful model for dementia palliative care in the community, it is essential to assimilate the perspectives and experiences of those affected. The guiding research question for this study was: What are people with dementia and family carers’ views on a model for dementia palliative care?.

**Methods:**

Focus groups (*n* = 3) were conducted with bereaved or current family carers (*n* = 11), and people with dementia (*n* = 2). Discussions centred around a proposed model of dementia palliative care. These were transcribed and analysed using thematic analysis.

**Results:**

Three main themes were identified: living and dying well with dementia; reducing carer burden to fulfil the wish for home care; and lack of faith in the healthcare system. One statement which summarised the analysis was: “Dementia palliative care is a dream, but not a reality.” This reflected participants’ repeated “wish” for this “ideal” model of care, but simultaneous scepticism regarding its implementation, based on their prior experiences of healthcare services.

**Conclusion:**

All participants were welcoming of the proposed model for dementia palliative care and were generally positive about palliative care as a concept relating to dementia. There was consensus that the model would allow people to live and die well with dementia, and reducing the carer burden would fulfil the wish to remain at home. However systemic changes in the healthcare system will be needed to facilitate a truly person-centred, holistic, individualised and flexible model of care.

## Introduction

Dementia is a syndrome with an ever-growing population. It is estimated that 55 million people globally live with one or more types of dementia, and with nearly 10 million new cases every year, it is the seventh leading cause of death in the world (World Health Organization, 2023). Dementia is caused by several different diseases and affects each person differently. Cognitive and non-cognitive symptoms include memory loss, reasoning and communication challenges, personality changes and a reduction in the ability to complete daily activities (National Institute for Health and Care Excellence, 2018).

An approach originally associated with cancer, palliative care can improve quality-of-life for all people with life-limiting illnesses, and their families, by addressing physical, psychosocial and spiritual needs (World Health Organization, 2020). While it is now recognised that people living with dementia can benefit from palliative care (Fox et al., 2020), they don’t have equal access to effective palliative and end-of-life care (Dempsey et al., 2015; Harrison Dening, 2016). The European Association of Palliative Care (EAPC) white paper (van der Steen et al., 2014) on optimal palliative care for older people with dementia highlighted key domains: applicability of palliative care; person-centred care, communication and shared decision-making; optimal treatment of symptoms and providing comfort; setting care goals and advance planning; continuity of care; psychosocial and spiritual support; family care and involvement; education of the health care team; societal/ethical issues; prognostication and timely recognition of dying; avoiding overly aggressive, burdensome or futile treatment (van der Steen et al., 2014). Core components of a model for palliative care for people with dementia living at home have also been put forward by service providers (Fox et al., 2020).

A study of bereaved carers (Mogan et al., 2022) highlighted the disparity between the consensus on the core aspects of end-of-life care (Poole et al., 2018) and peoples’ lived experience. Carers who had looked after a person with dementia at home during the last six months of life experienced: poor continuity of care, lack of expertise, limited advance care planning, and a loss of autonomy (Mogan et al., 2022). The importance of autonomy and treating the person with dementia with dignity and respect has emerged in several studies involving people with dementia and families about their perspectives on end-of-life care (Davies et al., 2017; Dening et al., 2013; Mogan et al., 2022; Russell et al., 2008).

People with dementia and their family carers concur that certain components are essential to end-of-life care: being cared for in place, being comfortable at the end-of-life, and having a skilled care team (Poole et al., 2018), with these views mapping to the EAPC domains. However, some differences of opinion exist (Poole et al., 2018). In a qualitative study of people with early-stage dementia and current or bereaved carers, carers put more value on future planning, prioritised decision-making on daily care issues, yet they felt poorly equipped to manage end-of-life care. On the other hand, people with dementia attached little value and importance to future planning, prioritised decision-making on medical care, and assumed their family would be capable of coordinating their end-of-life care (Poole et al., 2018). People with dementia have limited insight into the existing burden on carers, viewing burden as something that might happen in the future (Dening et al., 2013). Family carers need support around end-of-life as they manage the challenging dual role of being a grieving family member and a decision-maker (Hennings et al., 2010; Mogan et al., 2022).

Generally, the preference of people with dementia and family carers is to be supported to live and die at home, with family carers concerned about the person with dementia being left alone or isolated in other settings, and keen for them to have opportunities for social engagement and physical contact (Kupeli et al., 2016; Seow et al., 2023). While family carers generally agree with healthcare professionals on what’s important for end-of-life care for people with advanced dementia, an additional theme identified by family carers was for end-of-life care to be provided “at home” or in a “home-like environment” (Kupeli et al., 2016). From the family carer’s perspective, a suitable physical and social environment is key to achieving an adequate quality-of-life for the person with dementia (Russell et al., 2008).

To fully address the needs of palliative care for people with dementia, a unified, interdisciplinary and evidence-based approach is required that links from the local to national and global policy levels, informed by priorities and guided by frameworks and models of care (Fox et al., 2017). The Model for Dementia Palliative Care project sets out to develop an acceptable, evidence- and practice-based model for palliative care for people with dementia living in the community in Ireland (Developing a Palliative Care Model for People with Dementia in the Community, 2023). A draft model was developed using a programme theory approach and was presented as a logic model linking programme inputs to components and intermediate and long-term outcomes, while taking contextual and external factors into account and ensuring all elements translated into a model relevant to the community setting (paper under review). An important aspect of model development involved sharing drafts with stakeholders, including groups of people with dementia and family carers, to obtain critical feedback and iterate the model design.

The objective of this study is to examine the perspectives of a specific cohort (people with dementia and family carers) on a model of dementia palliative care in an Irish context. Their lived experience of accessing any of the proposed model components, and other insights into the proposed model from their broader experiences with dementia, help evaluate the proposed model, including the identification of any omitted areas of importance to them. The guiding research question for engagement with these groups was: What are your views on our model for dementia palliative care?

## Methods

### Design

A qualitative design was employed, using in-depth focus groups comprising people living with dementia and family carers. The discussion was framed in the context of the draft model for dementia palliative care in Ireland (see supplemental file 1). The draft model was shared with participants in advance by email, along with detailed written explanation of each component. During the focus groups, following initial rapport building questions, the six components of the model were presented: comprehensive care, person-centred practice and care, integrated care, accessible care, care for carer/supporter, and end-of-life care (more fully explained in Table 1). Each component was discussed sequentially with the key principles narrated by the facilitator (SF), along with examples of what the component might look like in practice (see detailed excerpt from one of the six components in Table 2). After each component was presented by the facilitator, an open discussion ensued, allowing participants to share their views on each component. The conduct and reporting of this study adhered to the COREQ guidelines (Tong et al., 2017).

**Table 1.** The six components of the model of palliative care for dementia with the features of each as a brief description.

Component		Brief description
1	Comprehensive Care that covers all dementia needs from diagnosis onwards	• Needs based, across 4 domains of palliative care
		• Includes non-medical and complimentary therapies
		• Proactive symptom assessment and management
2	Person Centred Practice and Care (PCC) that tailors the person’s care to their interests, abilities, history, personality	• Respectful and dignity-supporting
		• Co-design OR self-directed care
		• Patient passport
		• Decision-making & Advance Care Planning
3	Integrated Care that links people with services that work seamlessly together	• Community, Dementia and Specialist Palliative Care (SPC) teams working together, whenever necessary
		• Teams have the range/skillset to meet needs
		• Single point of contact for person with dementia/family
4	Accessible Care that ensures a basic level of support to everyone regardless of location, time, illness, ability	• Flexible home care hours
		• Equitable access to care based on needs
		• Responsive to changing needs over time
		• Pathway for urgent review
5	Care for Carer/Supporter ensures that family members are enabled to support the person with dementia and that their own needs are met	• Supported around decision-making processes
		• Information, Education and Training
		• Effective respite/restorative care
		• Bereavement/grief care (pre and post death)
6	End-of-Life Care ensures a dignified death in the person’s preferred place with limited suffering	• Recognition of dying, where possible
		• A comfortable environment
		• Excellent symptom management

**Table 2.** Detailed excerpt from component 1 “comprehensive care”.

Component 1: Comprehensive care that covers all dementia needs from diagnosis onwards
Key Principles
• A palliative care approach is applicable from diagnosis in dementia
• Goals of care include improving quality of life, maintaining function and maximizing comfort, and a different emphasis may be placed at different times
• Comprehensive care addresses all treatment and care in dementia, including for non-cognitive symptoms of dementia, and for both comorbid diseases and dementia-related symptoms
• The 4 pillars of palliative care are physical, psychological, social, and spiritual
o Good symptom management is key, with a holistic approach. Psychological, social, spiritual and alternative therapies etc are included (i.e., pharmacological and non-pharmacological interventions considered)
o Psychological and emotional support is critical. People with dementia might particularly need emotional support around the time of diagnosis and in early-stage disease. Families might need more emotional support as the disease progresses or at times of transition to higher care needs
o Social support to promote inclusion for people with dementia is part of holistic care, particularly in earlier stages, and to support family at all stages but particularly as the person with dementia’s condition advances
o Religious affiliation or sources of spiritual support should be assessed, and appropriate interventions (dignity therapy, multisensory stimulation), counselling (priest or spiritual counsellor) or rituals (e.g., prayers or hymns/songs) offered where appropriate
What might this look like in a service?
• Regular discussion and agreement about current/future goals of care with the person with dementia and carer/supporter. *[At least annually, more frequently as status changes]*
• Regular needs assessments conducted by a member of the MDT, considering physical, psychological, social, and spiritual needs. *[At least annually, more frequently as status changes]*. A formal tool to assess palliative care symptoms specifically in a person with dementia should be considered, such as IPOS dem (integrated palliative care outcome scale for dementia- community version available)
• Use of appropriate tools to assess individual symptoms, particularly as dementia progresses, and including the carer/supporter’s insight in such assessments *[e.g., for pain: PAIN AD or Abbey Pain scale; for mood: Cornell Scale for Depression in Dementia. Appropriate tools resource should be available nationally]*
• Availability of a range of pharmacological and non-pharmacological treatments to address symptoms *[Will depend on local resources, e.g., Reiki or massage for agitation]*
• Access to counselling and bereavement support services for the person/dyad.

### Sampling and recruitment

Inclusion criteria were broad: a person living with dementia, or a (current or previous) family member of a person living with dementia. We excluded people aged under 18 years and people not resident in Ireland. As recruitment required potential participants to read a plain language study information sheet, and opt-in to the research, it was unlikely that people with advanced dementia would take part, although they were not precluded from doing so. Using convenience sampling, participants were recruited through the Alzheimer Society of Ireland (ASI). A study poster and information sheet were circulated via email to members who expressed an interest in hearing about research studies. A list of those expressing interest was gathered by the ASI and shared with the research team who then made direct contact, answered any further questions, and arranged times for participation.

### Data collection

All focus groups were conducted by a female senior postdoctoral researcher experienced in dementia and qualitative research (SF). The focus group discussions took place online during March 2022 via videoconferencing software and with permission were digitally recorded. The recordings were transcribed verbatim with potential identifiers removed to uphold anonymity. Files were saved to an encrypted, password protected drive to ensure confidentiality.

A total of 13 participants took part in three focus groups (Table 3). Participants were given several potential dates, and selected to take part in the focus group that was most convenient for them. The focus groups opened with general introductions and discussion to build rapport. Focus groups lasted between 91–106 minutes (average 96 minutes). Two of the focus groups included a person with dementia (both with a diagnosis of young onset dementia, and both at early stage), while the other 11 participants were family carers. The family carer relationship to the person with dementia was either daughter (*n* = 8) or wife (*n* = 3). In some cases, the family carer’s loved one had passed away, and in others they were either living with the family carer or in long-term care. Of the sample, 92% were female, with only one male participant who was a person with dementia. All participants were resident in Ireland, living across various urban and rural settings. Participants were not required to have prior knowledge or experience of palliative care to be eligible for inclusion but were asked about this at the beginning of the focus groups. Participant’s familiarity with palliative care varied from no prior experience to one participant who previously worked as a paediatric palliative care nurse.

**Table 3.** Focus group participants’ demographics.

Participant ID	Relationship to person with dementia	Current carer or bereaved carer	Participant gender	Focus group no.
Person with Dementia-1	Self	N/A	Female	1
Family Carer-1	Daughter	Bereaved	Female	1
Family Carer-2	Daughter	Bereaved	Female	1
Family Carer-3	Daughter	Current	Female	1
Family Carer-4	Wife	Current	Female	2
Family Carer-5	Daughter	Current	Female	2
Family Carer-6	Wife	Bereaved	Female	2
Family Carer-7	Daughter	Bereaved	Female	2
Person with Dementia-2	Self	N/A	Male	3
Family Carer-8	Daughter	Current	Female	3
Family Carer-9	Wife	Current	Female	3
Family Carer-10	Daughter	Bereaved (mother)	Female	3
		Current (father)		
Family Carer-11	Daughter	Current	Female	3

### Data analysis

Qualitative data was coded and analysed in accordance with seven stages of thematic analysis (Braun & Clarke, 2013): 1. Transcription, 2. Reading and familiarisation, 3. Coding, 4. Searching for themes, 5. Reviewing themes, 6. Defining and naming themes, 7. Writing – final analysis. The process was managed with Microsoft Word and Excel software packages. The transcriptions were first read through to gain familiarisation with the content, with brief written memos used to record items of potential interest. Coding of the full dataset was undertaken independently by two researchers (SF and MF) before comparing the proposed codes. Codes relevant to the research question were applied, at a semantic level. The coding was then used to develop provisional themes and sub-themes, with particular attention given to identifying both positive and negative examples for each theme. The researchers consulted regularly, and after an iterative review of codes, themes and sub-themes, the final themes were defined before proceeding to completion of the analysis and final write-up (agreed by all authors).

### Ethical considerations

Appropriate guidelines for conducting psychosocial research with people with dementia were followed, including the Alzheimer Europe position paper (Gove et al., 2018). Written informed consent was obtained from all participants. Ethical approval was granted by the local Research Ethics Committee (ref: 2019-077).

## Results

**Figure 1.**
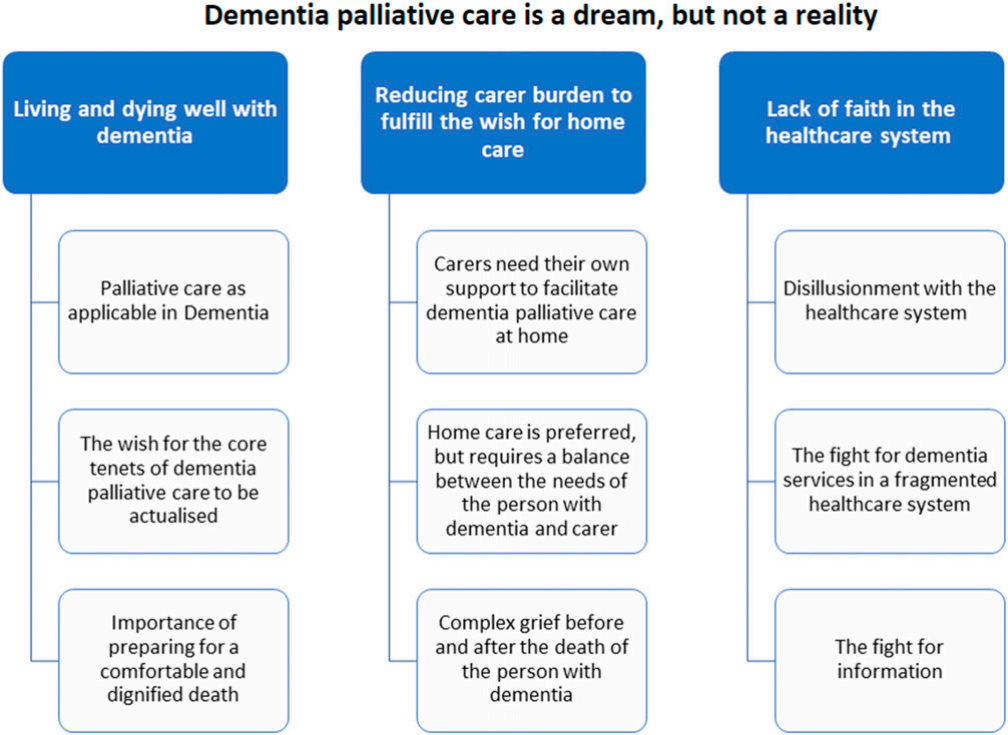
Thematic map of overarching theme, main themes, and sub-themes.

Overall, three main themes and nine sub-themes were identified in the dataset, relating to our guiding research question: What are people with dementia and family carers’ views on a model for dementia palliative care? The overarching theme summarising the analysis was expressed as follows: “Dementia palliative care is a dream, but not a reality.” This underscores participants’ repeated language around the “wish” for this “ideal” model of care, but their doubt that it could be implemented based on their poor prior experiences of services. A thematic map is presented in Figure 1.

### Theme 1: Living and dying well with dementia

#### Palliative care as applicable in dementia

All participants were welcoming of the proposed model, agreeing that it would allow people with dementia and their families to live and die well, and were generally positive about palliative care as a concept relating to dementia. One bereaved carer (Family Carer-6), whose husband had dementia, experienced specialist palliative care in a psychiatric hospital for the final six months of his life and described the care as “wonderful”. Others with experience of specialist palliative care through family members, or those with professional experience, recognised the importance of early palliative care for an improved end-of-life experience.

Participants said that the model represented what they wanted and needed. Some described it as a “wish list” for ideal care. One participant said it was upsetting to see the model written down as it is exactly what they wanted, but so removed from their actual experience.

While participants felt that palliative care is applicable to dementia, they perceived a wider stigma in society. They felt a wider public understanding of palliative care and openness to talking about death and dying would benefit everyone, including those living with dementia.

#### The wish for personalised, holistic, flexible care

Participants felt the person-centred care focus of the palliative care approach for dementia would greatly improve overall care. Participants agreed that the ultimate goal of dementia care, and in particular dementia palliative care, was to have the best quality-of-life possible, with their personhood respected:

“it’s a lot of behavioural issues Mom has, she doesn’t need a nurse, a doctor, she needs someone who cares and is going to put music on and rub her head and calm her down when she’s stressed and love her and that’s what she needs.” Family Carer-3

Relatedly, the holistic principles of the model were welcomed, in particular consideration of non-physical needs. A common discussion was the need for counselling for the person with dementia and/or their family members. Some family members found that while counselling was accessible to them, it was less accessible for the person with dementia. Participants also felt that health and social care practitioners (HSCPs) should take a more holistic approach to care; simply asking them about how they were feeling and coping, would greatly improve their experience and outcomes.

“I think just have somebody to listen to [the person with dementia] so they could get information on how they felt ... sometimes people just rush in and out of appointments, they don't really talk to them and nobody really listens to them” Family Carer-7

The groups agreed that an effective model of dementia palliative care needs to be individualised and flexible:

“If you’ve ... twenty people with Alzheimer’s ... every single one of those people is going to need different care, different hours, different times, and they can have the same dementia, be the same age, but their stages will be different, ... their needs will be different, their carers needs will be different, and it has to be individual care tailored to everyone” Person with Dementia-2

The need for care and settings that are appropriate to the individual’s personality and preferences were discussed:

“[my mother] isn’t suddenly going to start doing day care because it wouldn’t have been her personality whereas my dad did enjoy that now” Family Carer-10

Participants recounted the negative impact of receiving care that was inflexible and misaligned to their needs, such as home carers arriving early in the morning when the person with dementia was still asleep. Carers who were working full-time were exasperated with the inflexibility of the healthcare system:

“when my mum was in hospital... you’d be speaking to the nurses and [they’d say] ‘oh the doctors will be doing their rounds at 11:00 o’clock if you want to pop in’, like you can't just walk out of work and pop into the hospital on the off chance that somebody might be there to talk to you” Family Carer-7

Participants identified a significant gap in current services as their unavailability during evenings and weekends This left people feeling unsupported and precipitated potentially avoidable crisis events.

“not being able to contact the GP on a Friday evening…it just bugs me that people think you know nothing happens on Saturday and Sunday” Family Carer-2

#### Importance of preparing for a comfortable and dignified death

There was a consensus that while advance care planning (ACP) can be difficult and uncomfortable, it is hugely beneficial:

“[ACP] has taken a load off of my mind, it has taken a load off my wife’s mind, it’s taken a load off my kid’s mind. I think it’s of vital importance, the earlier the better because…it’s the people that matter made the decisions with me, and they know what I want, and I can’t stress how important that is” Person with Dementia-2

Many participants had never been engaged in a discussion about ACP by a HSCP, only learning about different care choices around end-of-life at a late stage.

“(husband’s name) went to hospital in February and it was then that the doctors raised it with us, you know, would you think about not coming back and I thought, oh my God, of course I would like I didn't know, I didn’t know that I could think about not coming back” Family Carer-6

Bereaved carers added that education around what to expect at end-of-life earlier in the course of the illness would have greatly helped them cope at end-of-life. Carers valued honesty around end-of-life, and HSCPs being forthright about what was happening. Knowing how much time the person had left ensures that the family have the chance to say goodbye.

“I think it’s very important that we know that it’s the end-of-life and that you know, people are honest with us” Family Carer-7

Fear of pain was a common thread in the conversations. Many participants expressed worry about pain at end-of-life for themselves or their loved one, especially as they feared a person with dementia can’t express their pain at advanced stages. One bereaved carer shared her experience:

“it still took a couple of weeks before the people in the nursing home recognised that she needed pain treatment i.e., morphine and I kind of wish that she could have had that morphine maybe two months beforehand and things would have been a lot easier for her and for the family as well” Family Carer-7

More positive experiences tended to be those where there was better support including palliative care involvement:

“we were so blessed that the GP associated with the nursing home was fantastic and … [prioritised] … that she wouldn’t be in pain, I mean my mum was nonverbal for the last two years of her life, so I mean literally you’re trying to judge from somebody’s eyes if they’re in pain … but I have to say … the nursing home was used to palliative care and … they dealt with it [managing pain] amazingly well” Family Carer-10

Key aspects of good end-of-life experience included being in a familiar, comfortable, and private environment; surrounded by loved ones; with their personhood and dignity respected:

“at the end of the day, all we all want just to be hopefully in our own homes but if that's not possible that we're comfortable, that we have a palliative care team there with us and to help our families and ourselves have a peaceful exit because there's nothing more than we want. Person with Dementia-1

Although assisted dying was not included in the model, it was raised as a key issue in two of the three focus groups. Participants in this group, including a person with dementia, were open to conversations around assisted dying and felt that it should be included in a model of dementia palliative care. Participants felt that it should be a choice for them and having the option would give them a sense of control over their illness and alleviate worries about pain.

### Theme 2. Reducing carer burden to fulfil the wish for home care

#### Carers need their own support to facilitate dementia palliative care at home

Carers spoke about the immense toll of caring for their loved ones with dementia. One person with dementia stated that the carers “*have it a lot tougher than the person with dementia” *(Person with Dementia-2). Carers made huge sacrifices such as moving home with young families from abroad, moving in with their loved one, or quitting work to care full-time. Many of the carers had little or no support; one spousal carer had only one “night off” in the previous three years. One carer living with her husband with a rare and progressive type of young onset dementia spoke about being “made to feel guilty” by society regarding her own needs and starkly outlined her “sacrifice” as a carer:

“I’m no longer living with the person I married, it’s a completely different person, I don’t know him, you know, and yet I have to do everything for him ... yet there’s no recognition of that sacrifice” Family Carer-9

Some carers felt aggrieved by a healthcare system that they felt didn’t consider their needs or provide the support they needed to care for their loved ones. They pointed out that carers are as individual as each person with dementia. For example, to facilitate them caring at home they need some respite, but at a time and duration that allows them to truly engage in something meaningful to them, to do “anything other than dementia” or “switch off”.

Most carers wanted to be the ones primarily caring for their loved one, but they felt to do this, they too needed care and support. Having overnight support was also important for carers wanting to support the person with dementia at home for end-of-life.

Some participants expressed a need to balance what the person with dementia wants and what the carer needs, particularly in the context of home care.

“it’s a battle between mom’s needs and what she deserves for her and what we’re able to do long term” Family Carer-3

One participant expressed her frustration at having no home support or GP support, and being left with no option but to bring her mother to hospital during a crisis, where the following exchange occurred:

“I brought my mother into A&E because I honestly didn’t know what else to do, and I was in a state, she was in a state...and then a doctor came up to me and said ‘d’you know the best place to care for someone with dementia is in the home?’ [laughs ironically] ... I didn’t bring her there because I didn’t want to care for her at home, I brought her there because I didn’t know what else to do!” Family Carer-7

#### Home care is preferred, but there needs to be other options

Overwhelmingly participants were supportive of a model which facilitates home care, and most family carers wanted to care for their loved ones at home, if possible:

“my mom ... I just can’t imagine putting her anywhere else because she deserves to be at home.” Family Carer-3

However, it appeared that an overemphasis on home care can lead to feelings of grief and “a sense of ... failure ... when the person can’t be at home” Family Carer-6

Overall, nursing homes were seen as sub-optimal care settings, and this perceived lack of suitable alternatives put pressure on carers to continue caring at home even when this was becoming unsustainable. Feelings of fear, regret and failure were expressed by some at the experience, or idea of, “putting” one’s loved one in a nursing home.

“nobody wants their loved one to be in a nursing home, particularly if it’s not something that they wanted themselves but sometimes it’s just not possible for the person with dementia to still be safely at home” Family Carer-1

However, one carer spoke about the very positive experience her father had in a specialist dementia long-term care facility.

“I left in tears when I dropped him in because I thought I'd just betrayed him... I but actually he had a great time … let’s maybe not throw out some of the existing models because it can work for people” Family Carer-1

Another reason for endorsing home care was the accessibility it offered. Participants spoke about the difficulty in physically accessing out-of-home services, needing to arrange time off work, transport, getting bloods done prior to an appointment. They would greatly prefer if care could come to them at home where possible (e.g. nurse comes to the house to take blood samples).

#### Complex grief before and after death of the person with dementia

Participants spoke of the often traumatic, slow decline in dementia, as one bereaved carer put it:

“I felt like I was losing [my mother] in the last few years of her life and it was kind of death by a thousand cuts” Family Carer-10.

It can be very difficult for carers who’ve devoted a significant time of their life to caring for someone to accept they are at end-of-life. One participant had found the following analogy helpful, likening the person’s decline towards end-of-life to *“letting go, not giving up”*.

It was evident in discussions that the carers who felt supported in their caring role were better able to cope following the death of their loved one. Carers who are entirely consumed with caring with no formal support can face a huge adjustment after the death of the person with dementia:

“a lot of people (carers) are tempted to give up their work and I would say don’t, do it if you can, if you could possibly manage without it because it’s important because after you know after the person’s gone” Family Carer-7

Good palliative care at end-of-life is important not only for the person with dementia, but also for the family as a bad experience can leave a carer with lasting trauma:

“If the person with dementia doesn’t have a good journey to the end the person that's left has to deal with that…” Family Carer-7

Those who had good palliative care support around end-of-life seemed less likely to suffer from complicated grief:

“when I knew that my mother didn't have long I was at peace in some ways because I just knew all I had to do was be with her and make sure she got the pain relief she needed” Family Carer-7

### Theme 3: Lack of faith in the healthcare system

#### Disillusionment with the healthcare system

While participants generally welcomed a model for dementia palliative care, they were disillusioned by their experience of the healthcare system thus far and were doubtful that the system could change to accommodate the model:

[regarding the dementia model of care]: “on paper looks fantastic but oh my god how you’re going to get to implement it I don’t know” Person with Dementia-2

A small number of participants had been involved with earlier initiatives, such as dementia training programmes, or dementia guidelines, and expressed frustration that these initiatives, while they were a source of great promotion and excitement, sometimes were not rolled out, or else failed to have much impact.

“The talking has got to stop and we’ve got implement these things” Person with Dementia-1

A few participants compared their experience of palliative care for loved ones with cancer with their experience of dementia care and were pessimistic about whether palliative care would be applied to them or their loved ones with dementia in the same way.

“my husband [who had cancer] would get every piece of care under the rising sun. I mean, I could not have asked for better healthcare than he got ... the people that I know that are at advanced stage of Alzheimer’s ... they don’t get the care, the families don’t get the care” Person with Dementia-1

#### The fight for dementia services in a fragmented healthcare system

A prominent theme underlying much of the focus group discussions was the “fight”, “struggle” or “battle” for dementia services. Geographic disparities were apparent.

“[Dementia services are] really on a demand basis, and by demand, I mean the people who shout the loudest and who are most persistent tend to be rewarded if it’s available in their area” Family Carer-1

Another carer spoke of only getting services as she “*played the system*” (Family Carer-5). Others felt that a situation must become very dire before help is offered:

“the prompt for [services] to get involved in the case of my mother, for example, was when she became a problem to them or to the system or to society” Family Carer-7

Participants were jaded and fatigued by their “fight”:

[regarding looking for services to keep the person at home] “I [am] fighting all the time, sometimes I can’t fight. I’m tired and all of us get tired of fighting.” Family Carer-3

The word “lucky” was used a lot when participants talked about services they did have, with some participants appearing embarrassed or apologetic mentioning that they had any services, knowing that others had no support at all. Another participant expressed that their father was “lucky” with the timing of his illness because there was support available as he neared end-of-life, whereas another family struggled to get support for their mother.

Some felt that cost was a personal barrier to them accessing services, but more felt that even if they were willing to pay privately for services, suitable care services are not available. Some participants were also sceptical of how elements of the model could work, when their experience was that many HSCPs in existing services are under-skilled in dementia care. Some recounted unsatisfactory experiences with GPs and hospital staff. Commonly, the group’s experience of home care staff was that they were not sufficiently trained in dementia care.

“we are paying about two and a half grand a week for carers and the carers are not as good as family. The reality is they don’t seem to have experience with Alzheimer’s or with dementia” Family Carer-3

#### Fight for information

Finding information was also framed as a “fight”. Participants felt that they often had to find information on their own, and sometimes came upon very useful information about a support or service only by chance. Carers, even those with backgrounds in healthcare themselves, found it very difficult to navigate the system in relation to dementia. They said that HSCPs rarely or never came to them with information about supports or services, that they had to figure this out on their own, adding to the stress they were experiencing.

“from a dementia point of view it’s still quite fragmented … no one person has all the information” Family Carer-5

Families would appreciate having a “one stop shop” or a named HSCP who knows them to coordinate their care, signpost them to relevant services, and provide information as needed, as is proposed in the model:

“The single point of contact is very important to have one person to coordinate everything” Family Carer-5

Others elaborated that this should be proactive management, where the contact person follows up with them. Family carers’ role as information providers was also framed as a fight; carers felt that they are a valuable source of information, but that they are not being listened to by HSCPs. Thus, while they welcomed the carer as being placed centrally in the model, they expressed some doubt as to how this would work in practice:

“[the model mentions] that the carer and supporters’ insights is valuable but how does that fit in with, you know, the medical profession and how they interact with us and confidentiality” Family Carer-7

There was also frustration expressed by many that the healthcare system is very fragmented with no facility to transfer information. Some worried that advance care plans or advance healthcare directives may not be followed in different settings.

“you go to different hospitals than you’re originally in and ... they don’t have the notes, they're starting from scratch” Family Carer-3

## Discussion

This study provides a detailed account of people with dementia and their families’ views on a proposed model of palliative care for people with dementia living at home in the community. The overarching theme was dementia palliative care is a dream, but not a reality. People with dementia and their family carers agreed that the core tenets of palliative care and dementia care, including care that is person-centred and holistic, delivered in an individualised and flexible way, represents their ideal care model. However, their unsatisfactory prior experiences of healthcare services led to scepticism that this model could be implemented.

Both family carers and people with dementia want good quality care at end-of-life including being cared for in place, being comfortable at end-of-life, and having a skilled healthcare team, which is consistent with views expressed in a previous study (Dening et al., 2013; Poole et al., 2018) and in the EAPC framework (van der Steen et al., 2014). Family carers want their loved one’s individual needs met, with personalised care that fosters respect and dignity (Davies et al., 2017). Themes of dignity and respect were common in previous literature (Davies et al., 2017; Dening et al., 2013; Mogan et al., 2022; Russell et al., 2008). This also speaks to the human rights argument of our shared humanness and the need for a culture of care and compassion (Cahill, 2018). In contrast to an earlier publication (Dening et al., 2013), people with dementia who participated here were acutely aware of the impact of their illness on their family carers, perhaps as these people had young onset and early stage dementia and were also involved in advocacy whereas the previous study included people with late onset dementia.

Engaging in ACP appeared to bring peace of mind to people with dementia and their families; previous research has associated ACP with better end-of-life outcomes (Dixon et al., 2018). However, as identified elsewhere (Mogan et al., 2022), many people with dementia and families had rarely been engaged in ACP by a HSCP or had limited knowledge of ACP (Seow et al., 2023) and there are barriers to undertaking ACP which need to be addressed, with the help of HSCPs (Dickinson et al., 2013). Fear of pain at end-of-life was common, and compounded by worries about the inability to communicate pain to others. This is consistent with other research which shows how important it is for families that their loved ones are pain-free, with carers feeling the only way to maintain control and independence was through assisted dying and euthanasia, but if end-of-life care was better, they would not need to contemplate euthanasia (Russell et al., 2008). Notably, assisted dying was discussed openly and frankly in our focus groups. This may have been influenced by current debate in Ireland around this issue, following the establishment of the Joint Committee on Assisted Dying in 2023. A model which would facilitate ACP, pain and symptom management and support grieving carers was greatly welcomed.

Literature supports the position that the preference of most people with dementia and their carers is for them to stay living at home in the community until end-of-life (Kupeli et al., 2016; Russell et al., 2008; Seow et al., 2023). As consistently outlined in the literature (Mogan et al., 2022; Russell et al., 2008), carers can experience immense burden while caring for loved ones, especially at end-of-life. This stress can be exacerbated when the views of people with dementia and family carers diverge (Poole et al., 2018), which reinforces the importance of supporting the family carer with care decisions. Carers felt they needed formal support to provide care at home. There was a feeling of pressure to provide care, with emotions of guilt, sadness and grief, when carers were not supported to do so, or they made a choice to move the person with dementia to another setting such as a nursing home, which was typically seen as a less preferable option. It should be noted that participants in the current study were all from a Western and typically individualised culture, and that there are cultural differences in preferences around palliative and end-of-life care (Ha, Lee & Yoo, 2023; Ha et al., 2023). Future work is needed to examine if minority groups have alternative needs within a model of dementia palliative care.

Irish healthcare frameworks appear to be consistent with a community model of dementia palliative care. The national Palliative Care model of care (2009) promotes that every person with a life-limiting condition, regardless of diagnosis, should be able to access a level of palliative care appropriate to their needs. The wider national health strategy in Ireland (“Slainte Care”) emphasises care at or near home, with equal access to health services for every citizen. Further, as far back as 2014 national policies have focused on dementia palliative care (Department of Health, 2014). However, a gap persists between policy and practice in the recounted experience of our participants.

While largely positive and welcoming of a model for dementia palliative care, participants were doubtful that it could be implemented. They were disillusioned by a healthcare system which made promises that were not kept and plans that weren’t implemented. This theme is consistent with a UK study, which found people with dementia and family carers had a lack of trust in medical decision-making (Dening et al., 2013). Participants had to fight for services, a theme also found in other research which highlights family carers’ struggle to navigate “unfamiliar territory” (10, p. 122), struggling with their lack of knowledge and experience, and poor communication with professionals; family carers had to fight for information and did not feel listened to, which was also a theme in other studies (Mogan et al., 2022; Smith et al., 2021). In Ireland, private home care is available, however even carers who could afford this found it unsatisfactory. Carers felt that HSCPs, particularly home care workers, were under-skilled in dementia care, as reported elsewhere (Mogan et al., 2022), manifesting in a lack of trust. Delivering person-centred dementia palliative care will require investment and support for creating a culture of care that is open to change.

## Conclusion

In this study, focus groups were conducted to answer the research question, what are people with dementia and family carers’ views on a model for dementia palliative care? Following analysis of a rich discussion, we can summarise that this cohort concur that dementia palliative care is very important and valuable, but they were disillusioned by their experience of the healthcare system and hence somewhat doubtful that a new model of care which is truly person-centred, individualised, and flexible, could become a reality. While consistent components of a model for dementia palliative care for people living at home have been agreed, cultural and systemic changes in the healthcare system and among the public more generally are needed to facilitate the implementation of a model of dementia palliative care that is acceptable to those who are directly affected by dementia. Key recommendations for implementing a model of dementia palliative care include: (i) garnering political support for the model of care and investing in changing societal attitudes, to de-stigmatise palliative care and dementia and facilitate open conversations about advanced illness and dying; (ii) exploring the necessary staff resources to deliver the required level of flexibility and tailoring and paid carer support at home, noting the high cost of the alternative (i.e. residential care) and the recent significant investment in dementia diagnostic and post-diagnostic support services in Ireland which provides opportunities to meet most needs with perhaps relatively modest extra resources; (iii) providing staff education and training to maximise the capacity of existing and new services to meet palliative care needs, such as emotional support, counselling, advance care planning. Implementing these recommendations will help bridge the gap between the current lived experience (“the reality”) and the ideal state (“the dream”) exemplified by the model of dementia palliative care.

## Supplemental Material

Supplemental Material - People with dementia and family carers are welcoming of a model of dementia palliative care, but sceptical of its implementation Supplemental Material for People with dementia and family carers are welcoming of a model of dementia palliative care, but sceptical of its implementation by Siobhan Fox, Jonathan Drennan, Mary Faherty, Suzanne Guerin, W George Kernohan, Aileen Murphy, Suzanne Timmons in Dementia.

## Data Availability

Raw data (i.e., interview transcripts) are not publicly available due to their potentially identifiable nature.*

## Appendix

### Abbreviations

ACP: Advance Care Planning
ASI: Alzheimer Society of Ireland
DPC: Dementia Palliative Care
EAPC: European Association of Palliative Care
GP: General Practitioner
HSCP: Health and Social Care Practitioner
MDT: Multi-Disciplinary Team
