# Supplementary figures and images for: People with dementia and family carers are welcoming of a model of dementia palliative care, but sceptical of its implementation
Source: Dementia (London). 2024 Aug 9;24(1):91–110. doi: 10.1177/14713012241270777 (PMC11667952; doi:10.1177/14713012241270777)

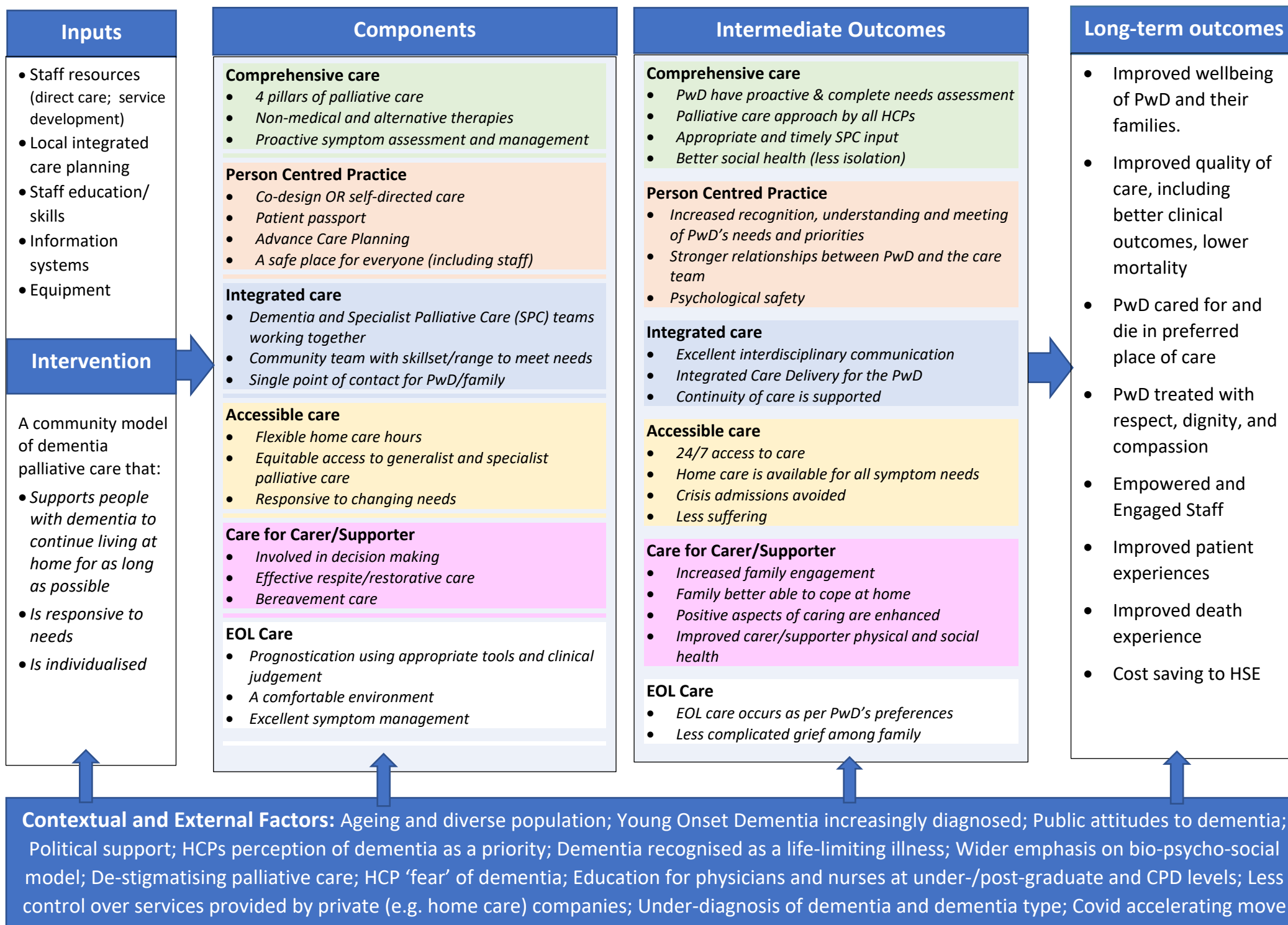

Supplement: Supplemental Material - People with dementia and family carers are welcoming of a model of dementia palliative care, but sceptical of its implementation [file sj-pdf-1-dem-10.1177_14713012241270777.pdf]
